# Susceptibility testing of *Anopheles* malaria vectors with the neonicotinoid insecticide clothianidin; results from 16 African countries, in preparation for indoor residual spraying with new insecticide formulations

**DOI:** 10.1186/s12936-019-2888-6

**Published:** 2019-08-01

**Authors:** Richard M. Oxborough, Aklilu Seyoum, Yemane Yihdego, Roch Dabire, Virgile Gnanguenon, Francis Wat’senga, Fiacre R. Agossa, Gedeon Yohannes, Sylvester Coleman, Lazarus Musa Samdi, Abdoulaye Diop, Ousmane Faye, Stephen Magesa, Alphaxard Manjurano, Michael Okia, Evelyne Alyko, Hieronymo Masendu, Ibrahima Baber, Arthur Sovi, Jean-Desire Rakotoson, Kenyssony Varela, Bernard Abong’o, Bradford Lucas, Christen Fornadel, Dereje Dengela

**Affiliations:** 1 PMI VectorLink Project, Abt Associates, 6130 Executive Blvd, Rockville, MD 20852 USA; 2 Institute of Health Science Research, Malaria and Tropical Neglected Research Unit, 01 BP 545, Bobo-Dioulasso, Burkina Faso; 3 PMI VectorLink Project, Abt Associates, Plot 28 Avenue Pierre Ngendandumwe, Bujumbura, Burundi; 4 Entomology Department, National Institute of Biomedical Research, Avenue de la Démocratie, Kinshasa, Democratic Republic of the Congo; 5 PMI VectorLink Project, Abt Associates, Kinshasa, Democratic Republic of the Congo; 6 PMI VectorLink Project, Abt Associates, Gerje Rood Sami Building, Floor 1, Office no 105, P.O. Box : 13646, Addis Ababa, Ethiopia; 7 PMI VectorLink Project, Abt Associates, Plot 11 Waterson Road, Fuo, Tamale, Ghana; 8 PMI VectorLink Project, Abt Associates, Gte No. 12, TOS Benson Crescent, Utako, Abuja, Nigeria; 9 PMI VectorLink Project, Abt Associates, Immeuble 8639, Sacré-Couer 2, Dakar Fann, PO Box: 25656, Dakar, Senegal; 10 Université Cheikh Anta Diop, Département de Biologie Animale, Bp 5005 Dakar-Fann, Dakar, Senegal; 11 PMI VectorLink Project, Abt Associates, PO Box 1212, Mwanza, Tanzania; 12 National Institute for Medical Research, PO Box 1462, Mwanza, Tanzania; 13 PMI VectorLink Project, Abt Associates, Tororo, Uganda; 14 PMI VectorLink Project, Abt Associates, Plot No. 3662, Njoka Road, Off Kwacha Road, Box 39090, Olympia, Lusaka, Zambia; 15 PMI VectorLink Project, Abt Associates, 1 Pascoe Avenue, Belgravia, Harare, Zimbabwe; 16 PMI VectorLink Project, Abt Associates, Ministry of Gender Compound, Capitol Bye Pass, Monrovia, Liberia; 17 PMI VectorLink Project, Abt Associates, Cite du Niger 1, Rue 30, Porte 612, Bamako, Mali; 18 PMI VectorLink Project, Abt Associates, Lot Ex La Sice, Ambalanaomby, Farafangana, Madagascar; 19 PMI VectorLink Project, Abt Associates, Rua Orlando Mendes, Nº183, Cidade de Maputo, Mozambique; 20 PMI VectorLink Project, Abt Associates, White House, Ojijo Oteko Road, Milimani Kisumu, Kenya; 21 U.S. President’s Malaria Initiative, U.S. Agency for International Development, Washington, D.C. USA

**Keywords:** Clothianidin, Neonicotinoid, *Anopheles gambiae*, SumiShield, Fludora Fusion, WHO susceptibility test, Diagnostic dose, Indoor residual spraying

## Abstract

**Background:**

In 2017, more than 5 million house structures were sprayed through the U.S. President’s Malaria Initiative, protecting more than 21 million people in sub-Saharan Africa. New IRS formulations, SumiShield™ 50WG and Fludora Fusion™ WP-SB, became World Health Organization (WHO) prequalified vector control products in 2017 and 2018, respectively. Both formulations contain the neonicotinoid active ingredient, clothianidin. The target site of neonicotinoids represents a novel mode of action for vector control, meaning that cross-resistance through existing mechanisms is less likely. In preparation for rollout of clothianidin formulations as part of national IRS rotation strategies, baseline susceptibility testing was conducted in 16 countries in sub-Saharan Africa.

**Methods:**

While work coordinated by the WHO is ongoing to develop a suitable bottle bioassay procedure, there was no published guidance regarding clothianidin susceptibility procedures or diagnostic concentrations. Therefore, a protocol was developed for impregnating filter papers with 2% w/v SumiShield™ 50WG dissolved in distilled water. Susceptibility tests were conducted using insectary-reared reference *Anopheles* and wild collected malaria vector species. All tests were conducted within 24 h of treating papers, with mortality recorded daily for 7 days, due to the slow-acting nature of clothianidin against mosquitoes. *Anopheles gambiae* sensu lato (s.l.) adults from wild collected larvae were tested in 14 countries, with wild collected F_0_ *Anopheles funestus* s.l. tested in Mozambique and Zambia.

**Results:**

One-hundred percent mortality was reached with all susceptible insectary strains and with wild *An. gambiae* s.l. from all sites in 11 countries. However, tests in at least one location from 5 countries produced mortality below 98%. While this could potentially be a sign of clothianidin resistance, it is more likely that the diagnostic dose or protocol requires further optimization. Repeat testing in 3 sites in Ghana and Zambia, where possible resistance was detected, subsequently produced 100% mortality. Results showed susceptibility to clothianidin in 38 of the 43 sites in sub-Saharan Africa, including malaria vectors with multiple resistance mechanisms to pyrethroids, carbamates and organophosphates.

**Conclusions:**

This study provides an interim diagnostic dose of 2% w/v clothianidin on filter papers which can be utilized by National Malaria Control Programmes and research organizations until the WHO concludes multi-centre studies and provides further guidance.

**Electronic supplementary material:**

The online version of this article (10.1186/s12936-019-2888-6) contains supplementary material, which is available to authorized users.

## Background

Insecticide-treated nets (ITNs) have been the primary form of malaria vector control undertaken in sub-Saharan Africa over the past decade. An estimated 1.04 billion nets were distributed in sub-Saharan Africa between 2009 and 2016, of which 582 million long-lasting insecticidal nets (LLINs) were distributed between 2014 and 2016 [1]. It is recognized that vector control has been the main driver in averting an estimated 663 million clinical cases of malaria in sub-Saharan Africa between 2000 and 2015, with ITNs contributing to 68% of cases averted and indoor residual spraying (IRS) to 11% [2]. While LLINs have been the most widespread vector control tool, IRS is another proven strategy, which in 2016 protected an estimated 45 million people in sub-Saharan Africa [1]. Despite the undoubted effectiveness of IRS in many transmission settings, the relatively high cost can be prohibitive and tends to limit implementation to relatively small target areas, often regions with the highest levels of malaria transmission [3, 4]. The US President’s Malaria Initiative (PMI) is one of the primary donors supporting IRS in sub-Saharan Africa through the PMI VectorLink Project (and previously the Africa Indoor Residual Spraying (AIRS) Project). In 2017, more than 5 million houses were sprayed through PMI VectorLink, protecting more than 21 million people in sub-Saharan Africa [5].

Insecticide formulations that currently have a World Health Organization (WHO) prequalification (PQ) listing for IRS belong to 4 chemical classes; namely carbamate, organophosphate, pyrethroid and neonicotinoid insecticides [6]. The organochlorine insecticide DDT (dichloro diphenyl trichloroethane) is also utilized in several countries, such as India and Zimbabwe but there is currently no DDT product with PQ listing [7]. This may make it seem like there are a plethora of options for insecticide rotation, however, before neonicotinoid formulations were PQ listed, there were only 2 modes of action across 4 insecticide classes, namely sodium channel modulation (pyrethroids and DDT) and acetylcholinesterase (AChE) inhibition (organophosphates and carbamates) [8]. This lack of product diversity increases the likelihood of cross-resistance between products with the same mode of action [9].

Many countries in sub-Saharan Africa have adopted the policies outlined in the Global Plan for Insecticide Resistance Management (GPIRM) produced by the WHO and have prepared national resistance management documents according to the WHO framework [10, 11]. According to several national policy documents, insecticide rotation should be undertaken in areas of IRS, using insecticides with different modes of action against susceptible malaria vectors [12–14]. However, resistance to DDT and pyrethroids is widespread and several countries have reported resistance to carbamates in some regions [15]. Pirimiphos-methyl resistance is also beginning to emerge in some settings [16]. In addition, the residual duration of IRS formulations is of key importance, with products such as the carbamate Ficam™ WP (wettable powder) showing extremely short residual duration on substrates such as mud, being unsuitable in areas with long transmission seasons [17]. The end result is that there have been few, if any, viable options for insecticide rotation. Therefore, despite having established policies based on GPIRM, countries have generally been unable to practically implement insecticide rotation for insecticide resistance management.

It has taken 40 years for new chemical classes to be developed for malaria vector control. Until the recent WHO listing of neonicotinoid and pyrrole insecticides, the pyrethroids were the last new chemical class to be brought through to the public health market, in the 1980s, and have proved to be highly successful both for LLINs and IRS [18, 19]. New IRS formulations, SumiShield™ 50WG (water dispersible granules) and Fludora Fusion™ WP-SB (wettable powder in a water soluble bag), became WHO prequalified vector control products in 2017 and 2018 respectively and were included to the list of products which meet Global Fund requirements for procurement [6, 20]. The active ingredient of SumiShield™ 50WG is the neonicotinoid insecticide, clothianidin, which is applied in IRS at a target dose of 300 mg ai/m^2^, while Fludora Fusion WP-SB contains a mixture of clothianidin and deltamethrin (pyrethroid) and is applied at 225 mg ai/m^2^. Neonicotinoid compounds were first developed for agricultural use in the 1990s and soon became the most widely used pesticides in the world against a broad spectrum of economically important crop pests [21]. Clothianidin (one of at least 8 registered neonicotinoid insecticides) is a metabolite of another neonicotinoid insecticide, thiamethoxam [22]. Both thiamethoxam and clothianidin are market leading broad spectrum, systemic compounds used in agriculture, which diffuse throughout the crop, including the roots and foliage, thus providing long-lasting protection against a variety of sucking and chewing pests [22]. While clothianidin is primarily a systemic insecticide targeting agricultural chewing pests, it can also be applied inside houses to control malaria vectors through tarsal contact. The target sites of neonicotinoids are nicotinic acetylcholine receptors (nAChR), which represent a novel mode of action for vector control, meaning that cross-resistance through existing mechanisms is unlikely [8].

Studies conducted in experimental huts in Benin showed that SumiShield™ 50WG produced high levels of *Anopheles gambiae* sensu lato (s.l.) mortality for at least 8 months [23]. While village scale IRS in India demonstrated control of *Anopheles culicifacies* for at least 5 months and was operationally accepted by spray operators and community members [24]. Fludora Fusion™ WP-SB has also shown similar impressive residual longevity in experimental hut trials in Benin, lasting for at least 8 months on mud and cement walls [25].

In 2017, all IRS programs funded by PMI in sub-Saharan Africa were conducted with Actellic™ 300CS (capsule suspension) in areas with pirimiphos-methyl susceptible malaria vectors. However, in many countries Actellic™ 300CS has been sprayed annually, for up to 4 consecutive years, and it is important to implement rotation strategies as soon as possible, before resistance develops. Baseline clothianidin susceptibility testing was conducted in 16 countries in sub-Saharan Africa to determine whether there was any resistance prior to rollout of SumiShield™ 50WG and Fludora Fusion™ WP-SB, as part of national rotation strategies.

## Methods

### Study sites

**Fig. 1:**
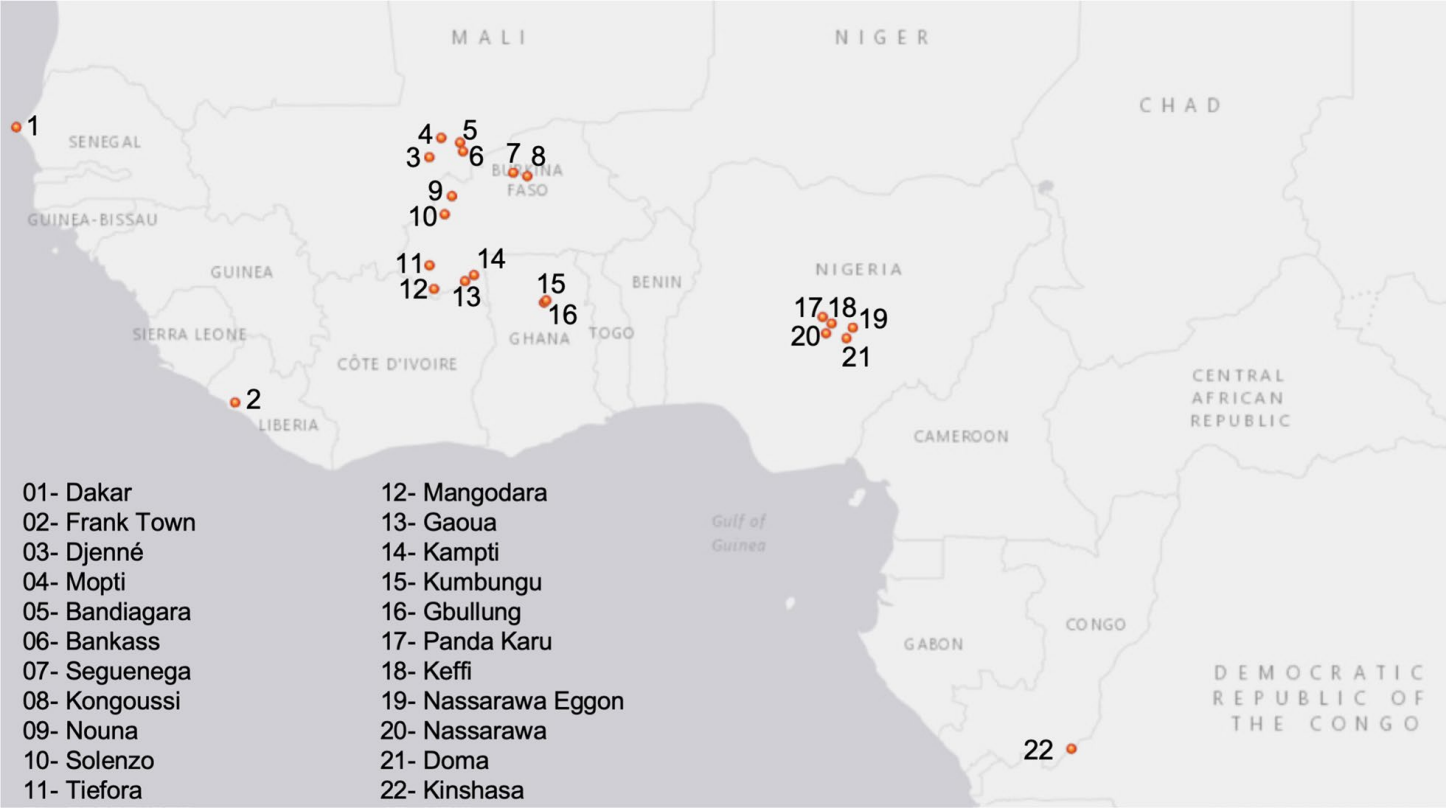
Locations in West and Central Africa where clothianidin susceptibility testing was conducted in 2016–17

**Fig. 2:**
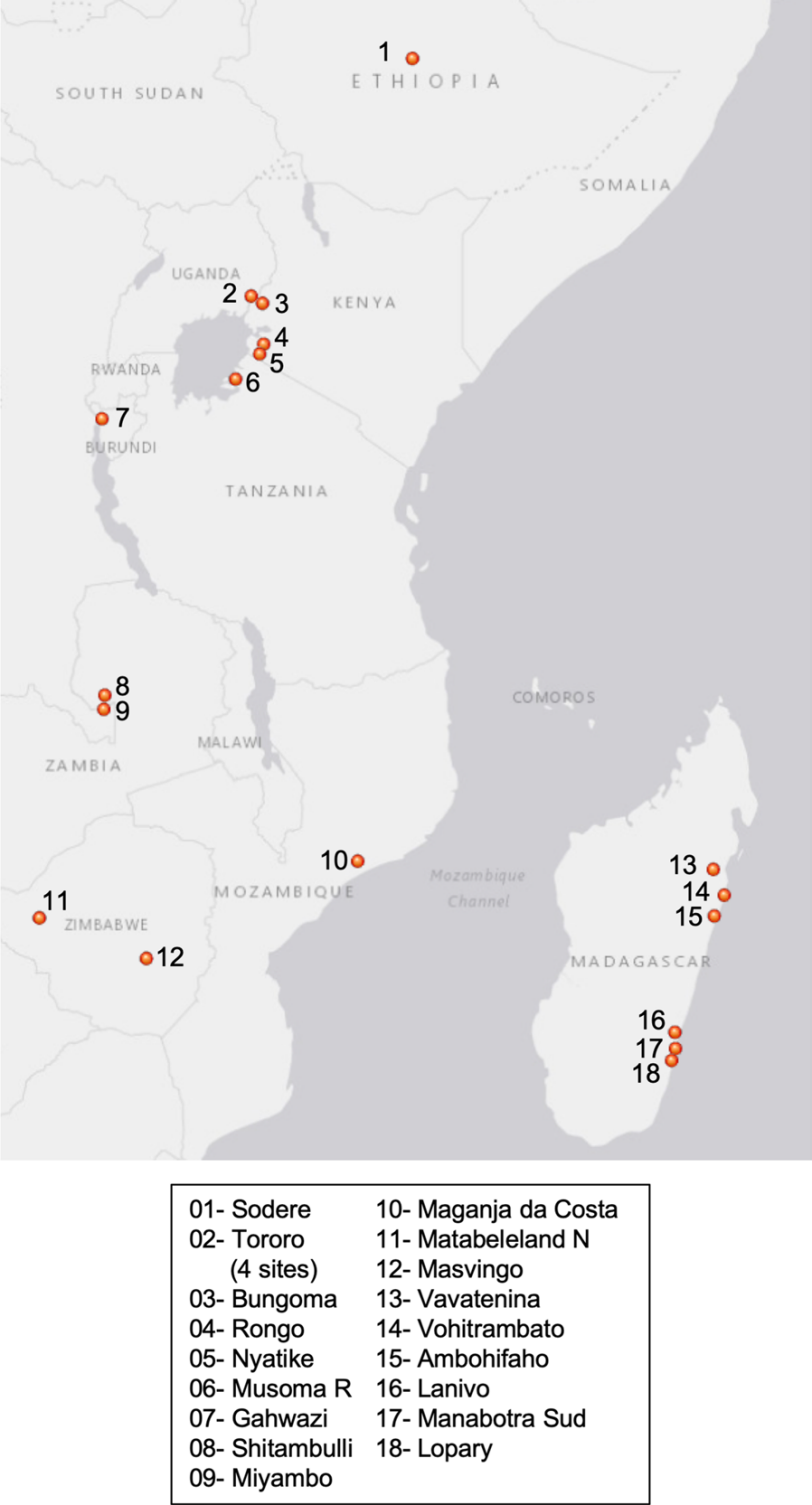
Locations in East and Southern Africa where clothianidin susceptibility testing was conducted in 2016–17

As part of the PMI VectorLink project, insecticide resistance monitoring is conducted annually in partner countries in sub-Saharan Africa with commonly used insecticides for malaria vector control. In anticipation of SumiShield™ 50WG (November, 2017) and Fludora Fusion™ WP-SB (December, 2018) receiving WHO PQ listing, PMI VectorLink entomologists and partners collected baseline data to determine vector susceptibility status to clothianidin. Each of the 16 countries conducted testing in at least one location using field collected *Anopheles* mosquitoes along with an insectary reared susceptible strain. Figure 1 shows the location of sites in West and Central Africa and Fig. 2 shows sites in East and Southern Africa where clothianidin susceptibility bioassays were conducted.

### Preparation of insecticide-treated papers

Insecticide susceptibility tests are normally conducted using pre-treated filter papers that are prepared by a WHO collaborating institution (Universiti Sains, Malaysia) and distributed to field sites [26]. Diagnostic doses are determined by WHO through testing of multiple mosquito species in different countries. However, as of 2019 there was no published guidance from WHO regarding clothianidin susceptibility test procedures or diagnostic concentrations. Therefore, a protocol was developed and optimized by Sumitomo Chemical Company (SCC). SCC conducted WHO cylinder tests to determine a suitable solvent and diagnostic dose for clothianidin susceptibility tests. A dose range of 0.125%, 0.25%, 0.5%, 1%, 2% and 4% w/v (weight per volume) clothianidin active ingredient were tested in WHO cylinder tests against 6 insectary strains of *An. gambiae* and *Anopheles arabiensis*. Acetone, hexane and water were tested as solvents. Results showed that both hexane and water were suitable solvents (poor results were obtained using acetone) for evaluating clothianidin in WHO cylinder tests and that 1% w/v clothianidin provided 100% mortality against all 6 strains (including pyrethroid resistant strains). The same result was observed using either technical grade clothianidin or formulated SumiShield™ 50WG. The diagnostic dose was set at 2% w/v clothianidin (i.e. twice the minimum dose that killed 100%), with water or hexane as suitable solvents (Ohashi, pers. commun.).

Therefore, in each site, filter papers were treated in situ with 2% w/v clothianidin (equivalent of 734 mg/m^2^), by treating each filter paper with 26.4 mg of SumiShield™ 50WG, diluted in distilled water. SumiShield™ 50WG was used instead of technical grade material, due to availability of formulated product. However, a limitation of using formulated material is greater batch-to-batch variation than technical grade active ingredient. Whatman filter papers (grade 1) were cut to 12 cm by 15 cm to be the same size as WHO filter papers used in tube tests. An insecticide solution was prepared by adding 264 mg of SumiShield™ 50WG granules to 20 ml distilled water in a Falcon™ tube and shaking until fully dissolved. The filter paper was supported on a bed of nails (which were hammered into a piece of wood at equal heights) during insecticide application, to allow for even absorption into the filter paper. A pipette was used to transfer 2 ml of insecticide solution evenly onto the filter paper by carefully dispensing rows of droplets until there were no dry sections of the filter paper at a target dosage of 13.2 mg/active ingredient per paper (see video [27]). Treated filter papers were left in a dark cupboard to dry overnight. As the stability of SumiShield™ 50WG on filter papers has not been established, all tests were conducted within 24 h of treating the papers.

### Insecticide susceptibility tests

Insecticide susceptibility tests were conducted according to established WHO protocols, with modifications made to the holding period [26]. In general, a total of 100 (depending on availability) *An. gambiae* s.l. or *Anopheles funestus* s.l. were exposed for 60 min in 4 replicates of 25 mosquitoes, with an additional 1 or 2 replicates of 25 mosquitoes used for the negative control (paper treated with distilled water). Results for the negative control bioassays are presented in supplementary file 1. After exposure, mosquitoes were transferred to clean holding tubes and provided with sugar solution. Mortality was recorded every 24 h after exposure for a maximum of 7 days, or until 100% mortality was reached. Tests were conducted in the morning and holding conditions were intended to be within WHO guidelines of 27 °C ± 2 °C and relative humidity of 75% ± 20%. Temperature and humidity were monitored, however, in most cases could not be accurately controlled, as tests with wild collected mosquitoes were generally conducted under field conditions. A summary of max/min temperature and humidity is available as Additional file 1: Table S1.

### Mosquito species tested

Tests were conducted using insectary-reared susceptible *Anopheles* as well as wild collected malaria vector species. Insectary-reared mosquitoes included susceptible *An. gambiae* sensu stricto (s.s.) Kisumu (Burkina Faso, Burundi, Ghana, Nigeria, Tanzania, Uganda, Zambia), *Anopheles coluzzii* Yaoundé (DR Congo, Mali, Senegal) and *An. arabiensis* KGB (Ethiopia and Zimbabwe), based on previous molecular analysis as part of routine insectary colony checks. Insectary colonies were not tested in Kenya, Liberia, Madagascar, and Mozambique.

Larval collections were made at various times between September 2016 and December 2017 for each study site using larval dippers. Larvae were subsequently transported to a field insectary for rearing, usually using field collected water, with larvae fed using Tetramin^®^ fish food. Emerging adult mosquitoes were provided with sugar solution until they were used in insecticide susceptibility tests when aged 2 to 5 days. Wild *Anopheles* were identified morphologically as *An. gambiae* s.l. in 14 of 16 countries, and wild caught F_0_ *An. funestus* s.l. adults of unknown age were tested in Mozambique and Zambia. Molecular species analysis of wild mosquitoes tested was not conducted.

### Data analysis

Insecticide susceptibility results were presented as percentage mortality every 24 h after bioassay exposure for a maximum of 7 days. If negative control mortality was greater than 20%, the data was discarded and tests repeated. Control mortality results are presented for all tests in Additional file 1: Table S2. WHO criteria were used for interpretation, with 98–100% mortality indicating susceptibility; 90–97% indicating possible resistance, with resistance genes to be confirmed; and mortality < 90% indicating resistance [28]. PoloPlus (LeOra Software) was used to conduct probit analysis on the logarithmic scale to calculate the time taken for a fixed concentration of clothianidin to kill a defined proportion of insects, known as lethal time (LT). For example, the LT_50_ is the lethal time predicted for 50% mortality to be reached.

## Results

### Tests with insectary-reared susceptible *Anopheles* colonies

**Fig. 3:**
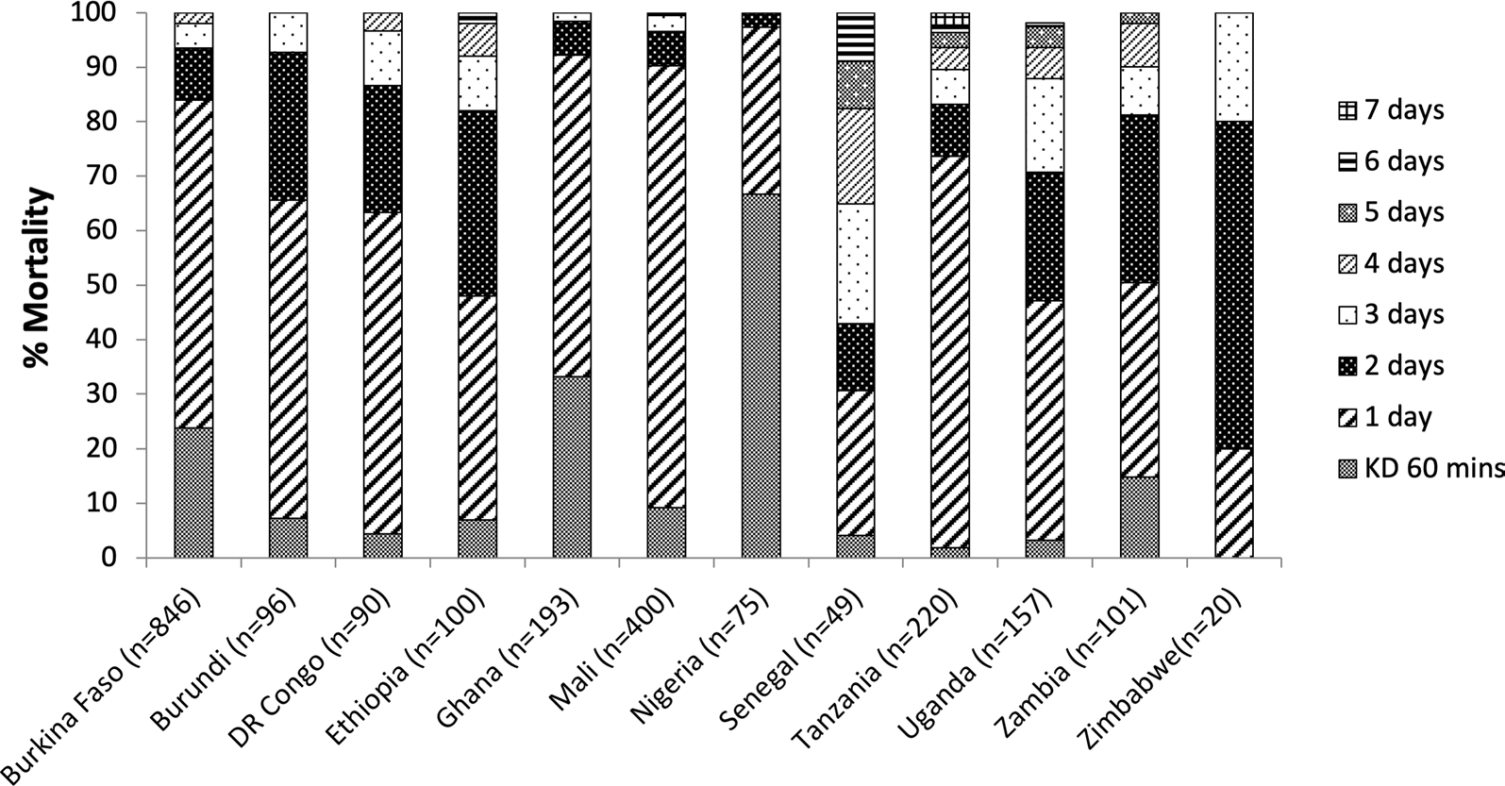
Percentage mortality of insectary reared susceptible *An. gambiae*, *An. coluzzii*, and *An. arabiensis* following 60 min exposure to clothianidin treated filter papers in WHO tubes in 12 countries

**Fig. 4:**
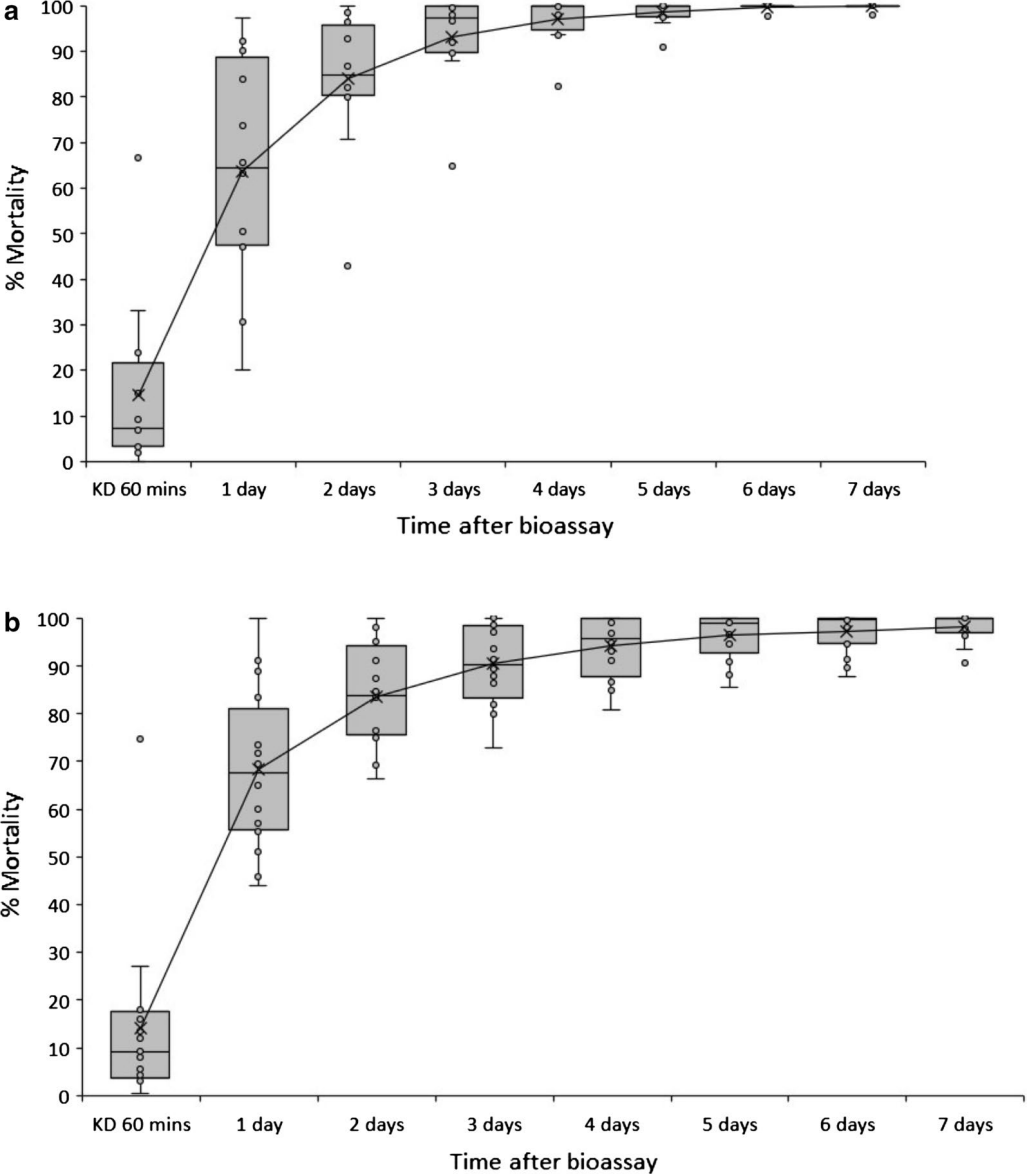
Box and whisker plot showing median and mean mortality and interquartile range following 60 min exposure to clothianidin treated filter papers in WHO tubes for: **a** Insectary reared susceptible *Anopheles* (combined data for *An. gambiae*, *An. arabiensis* and *An. coluzzii*) from 12 countries, and **b** wild collected *Anopheles* (combined data for *An. gambiae* s.l. and *An. funestus* s.l.) from 16 countries

Results are presented in Fig. 3 for 12 countries where clothianidin susceptibility testing was conducted using insectary reared susceptible colonies. Figure 4a represents results for the median, mean and interquartile range for 60 min knock-down and mortality every 24 h for 7 days after exposure. Knock-down proportions were generally low, in most cases < 20% after 60 min (Figs. 3, 4a). There was large variation in mortality rates 24 h after exposure, ranging from as low as 20% and reaching 100%. The interquartile range at 24 h was between 48 and 79% (Fig. 4a). Three days after exposure the vast majority of mortality had taken place, with an interquartile range of 90–100% (Fig. 4a). Between 4 and 7 days after exposure any increase in mortality was relatively low. However, in many cases holding for 7 days increased mortality up to 100%. Overall, 100% mortality was reached in all tests (except for 98% in Uganda) 7 days after exposure (Fig. 3).

### Tests with wild F_1_*An. gambiae* s.l. and F_0_*An. funestus* s.l

**Fig. 5:**
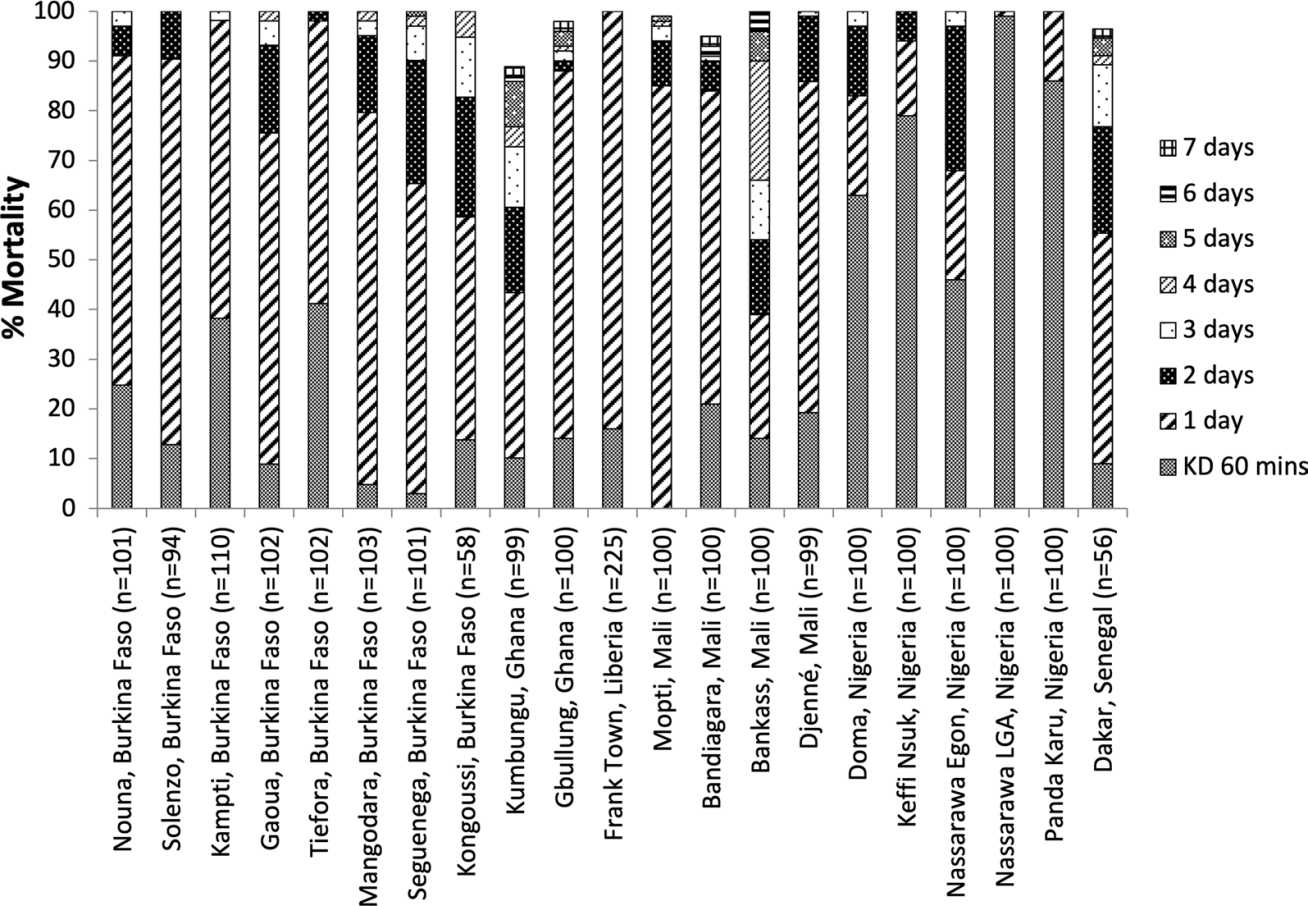
Percentage mortality of wild F_1_ *An. gambiae* s.l. from sites in West and Central Africa following 60 min exposure to clothianidin treated filter papers in WHO tubes

**Fig. 6:**
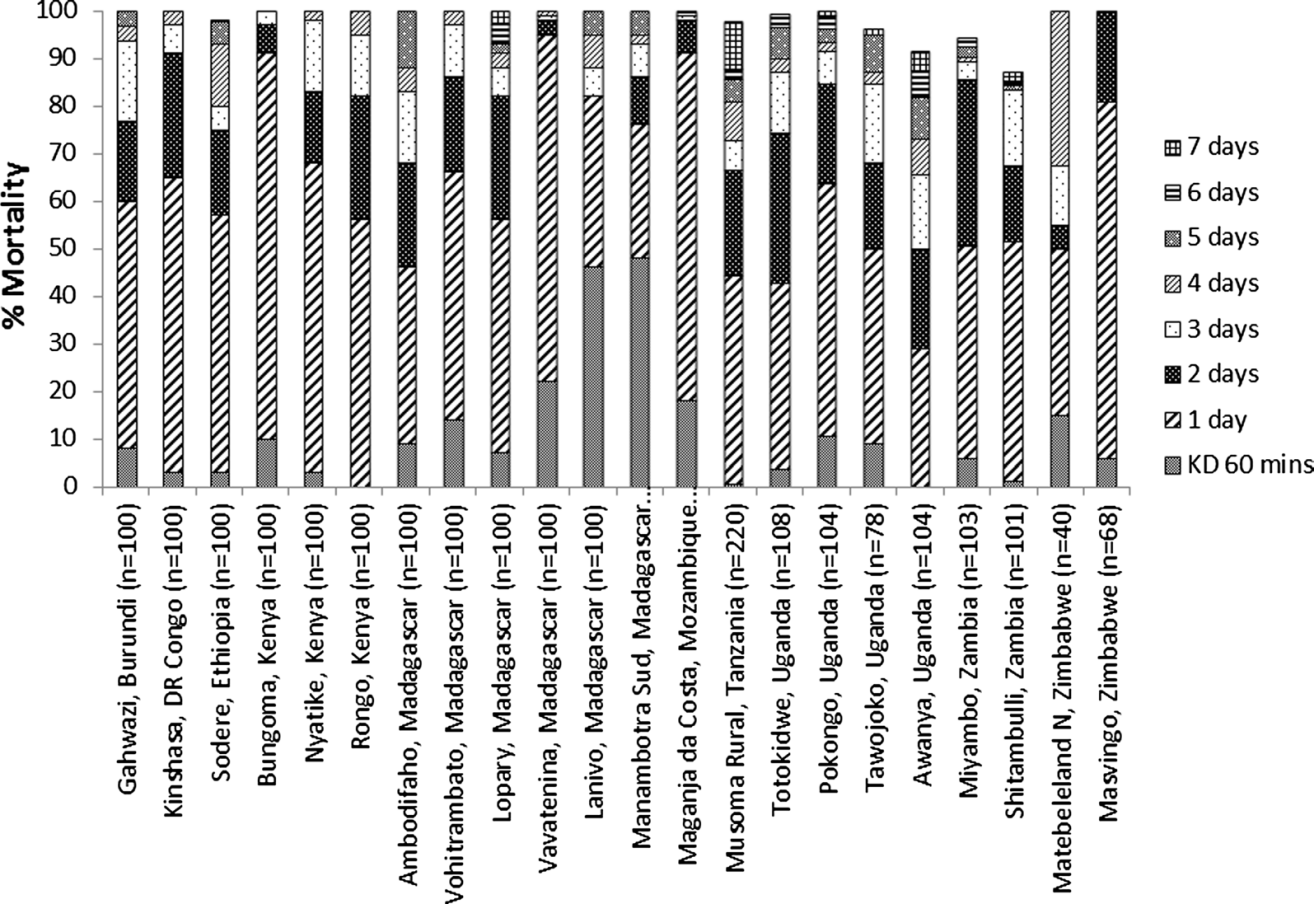
Percentage mortality of wild F_0_ *An. funestus* s.l. (Mozambique and Zambia) and F_1_ *An. gambiae* s.l. from sites in East and Southern Africa following 60 min exposure to clothianidin treated filter papers in WHO tubes

Figure 4b represents results for the median, mean and interquartile range with wild *Anopheles* from 16 countries. More detailed results are presented by site in Fig. 5 for sites located in west and central Africa and in Fig. 6 for sites located in East and Southern Africa. Overall, 11 out of 16 countries reported > 98% mortality in all sites tested, within 7 days of exposure, indicating susceptibility. Mortality at 7 days was 95% in Bandiagara in Mali, but in neighbouring districts of Mopti Region (Bankass, Mopti, Djenné) mortality was 100%. In Dakar, Senegal mortality only reached 96% after 7 days. Mortality after 7 days in Tororo Region of Uganda varied, with a minimum of 91% mortality in Awanya, but 100% in Nagongera. While in Kumbungu Region of Ghana, 98% mortality was reached in Gbullung, but only 89% in Kumbungu town after 7 days. In Zambia, mortality for *An. funestus* s.l. was 94% in Miyambo and 87% in Shitambulli after 7 days.

**Fig. 7:**
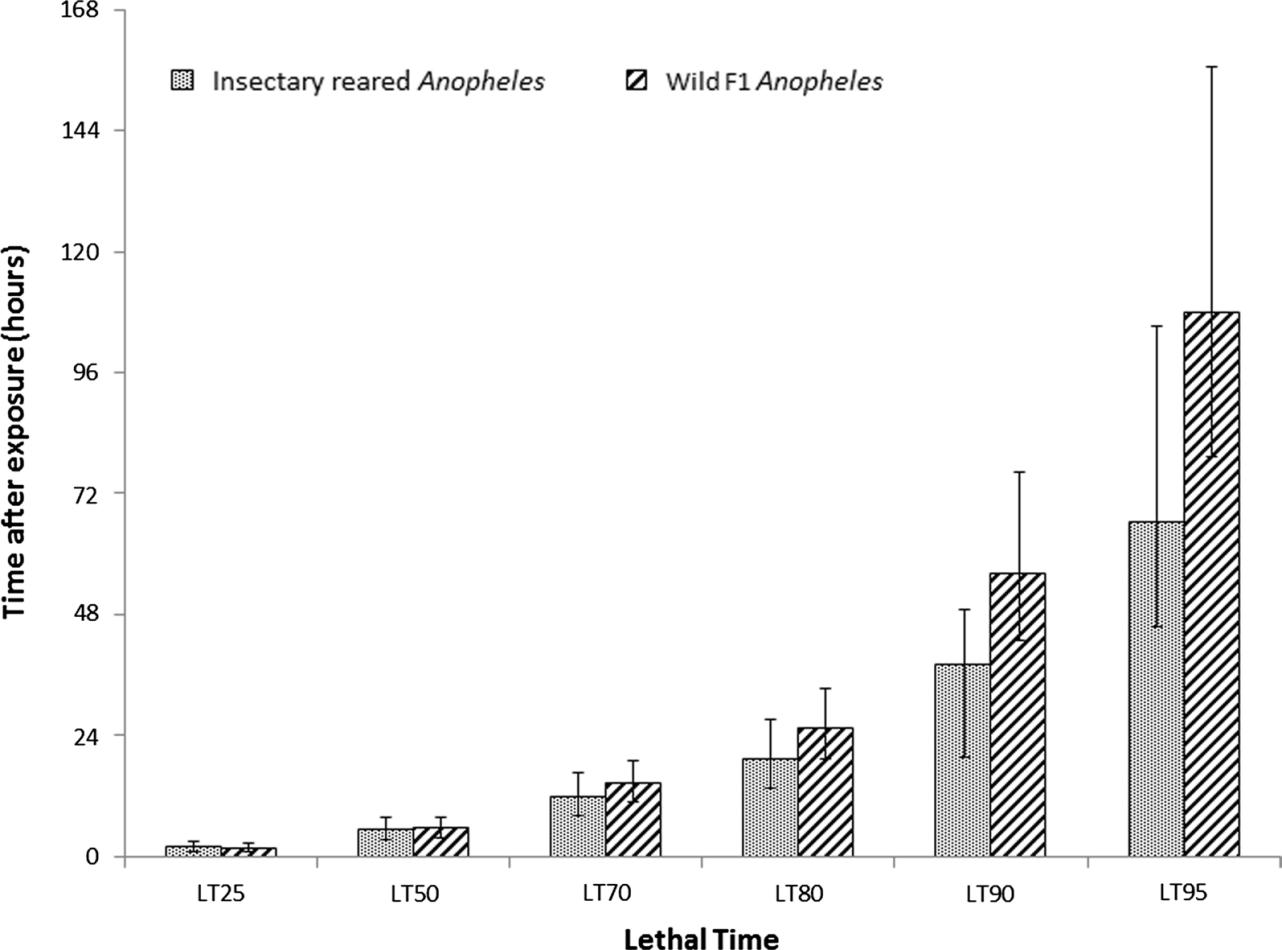
Probit analysis used to estimate the holding time, after 60 min exposure to a fixed concentration of 13.2 mg clothianidin active ingredient per paper, expected to result in 25, 50, 70, 80, 90 and 95% mortality

Results of probit analysis for wild collected *Anopheles* and insectary reared susceptible mosquitoes produced similar results, with a non-linear trend showing that the majority of mortality occurred within the first 24 h and 90% of mortality was estimated to take place 72 h after exposure (Fig. 7). There was minimal increase in mortality between 3 and 7 days after exposure.

### Repeat tests in sites where possible resistance was detected

**Fig. 8:**
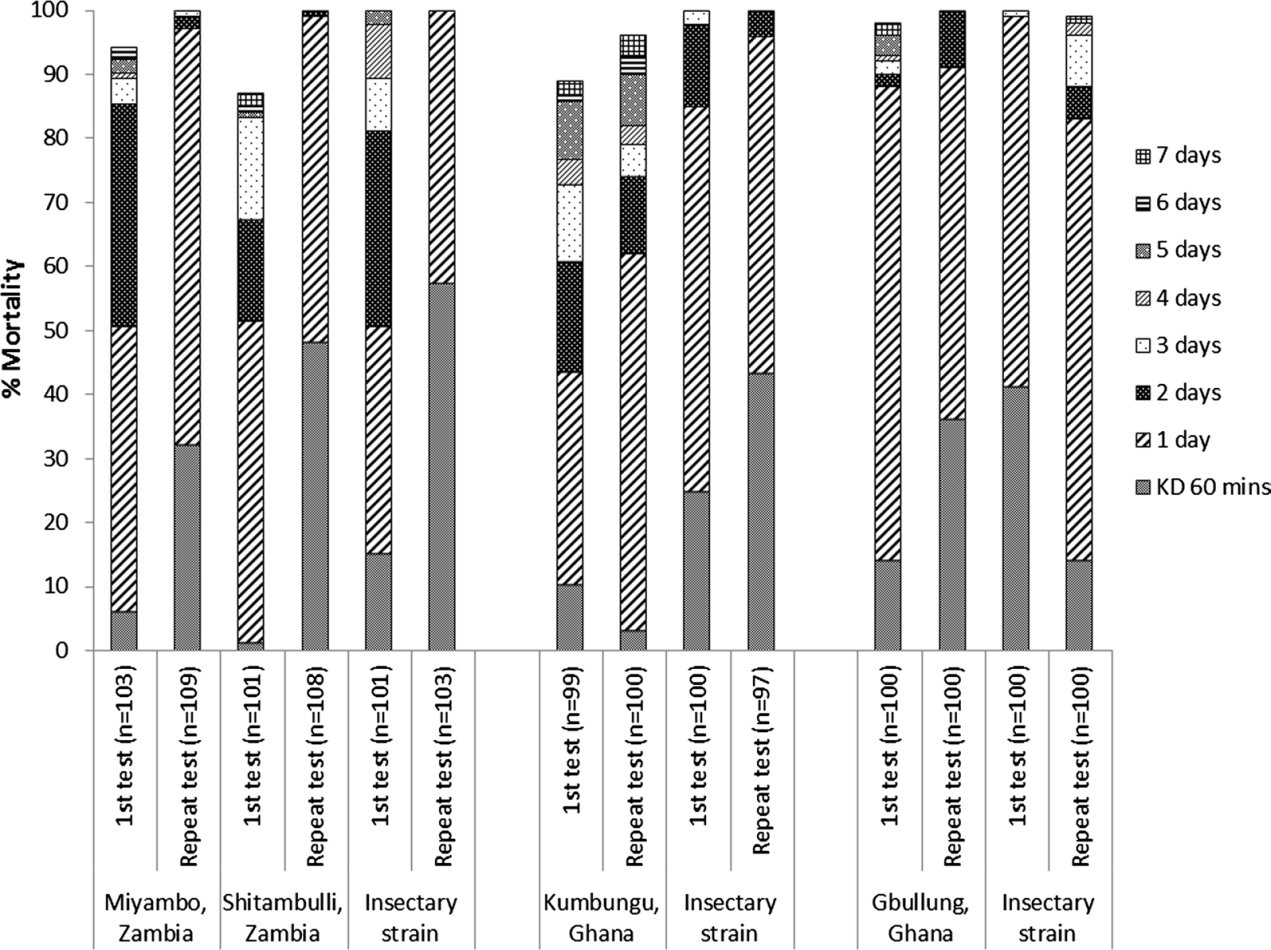
Percentage mortality of wild F1 *An. gambiae* s.l. and insectary reared *An. gambiae* (Kisumu) following 60 min exposure to clothianidin treated filter papers in WHO tubes, from repeated tests for sites in Zambia and Ghana

Susceptibility tests were repeated several months later, in some sites where possible resistance to clothianidin was recorded in the first round of tests. Repeated tests of wild *An. funestus* s.l. in Miyambo and Shitambulli villages, Zambia produced 100% mortality within 3 days of exposure (Fig. 8). Tests done in parallel with an insectary strain of *An. gambiae* Kisumu also produced 100% mortality but in a faster time, within 1 day of exposure. It should be noted that in the first tests where possible resistance was recorded in wild *An. funestus* s.l., the *An. gambiae* Kisumu strain was killed more slowly than in the repeat tests, with 100% mortality only reached 5 days after exposure. This may indicate differences in dosing between the filter papers used in the first and repeat tests. Repeat tests conducted in Ghana in December 2017 produced 96% mortality in Kumbungu and 100% mortality in Gbullung, compared to 89% and 98% respectively in the first round of tests. Parallel tests conducted in both villages with the *An. gambiae* Kisumu insectary strain were consistent and provided 99–100% mortality for the first and repeat tests.

## Discussion

Susceptibility bioassays conducted with insectary and wild *Anopheles* species confirmed that clothianidin is a relatively slow-acting insecticide, at the concentration tested, unlike other neurotoxic insecticides such as pyrethroids, organophosphates, carbamates and organochlorines. Generally more than 90% of clothianidin-induced mortality occurred within 72 h of exposure, however, 7 days was often required to reach 100% mortality. While standard WHO susceptibility bioassays have a holding period of 24 h after exposure, it was necessary to record delayed mortality for up to 7 days for clothianidin susceptibility tests. This creates greater demands on field entomology teams to keep control mortality below 20% for 7 days and also increases cost, as more time is spent in the field.

Full susceptibility was recorded with all susceptible insectary strains and > 98% mortality for wild *Anopheles* at all sites in 11 out of 16 countries. However, tests in at least 1 site in 5 countries produced mortality < 98%, which could potentially be a sign of existing clothianidin resistance. A more likely explanation is that the diagnostic dose protocol needs further testing and modification. Normally, the WHO diagnostic concentration is twice the lowest concentration that gives systematically 100% mortality, in order to avoid false reporting of resistance. In this case the diagnostic dose killed 100% of insectary strains but may have been too low to prevent false detection of resistance. Inbreeding over a period of several decades generally reduces the overall fitness of reference insectary strains [29]. Therefore, slightly lower levels of mortality when testing wild *An. gambiae* s.l. is expected and does not necessarily represent evidence of true resistance. Indeed, repeat testing in Ghana and Zambia, where possible resistance was detected, subsequently showed 100% mortality in 3 sites. This highlights the need to repeat bioassays to confirm resistance, particularly when insecticide-treated papers are being self-prepared and especially when resistance is being reported for the first time. It was also useful to test an insectary strain in parallel, using the same treated filter papers, as a way to determine whether self-dosing of papers was a potential issue. In Kumbungu, Ghana, parallel testing with a susceptible insectary strain produced 100% mortality, while mortality was only 89% and 96% in 2 tests with wild *An. gambiae* s.l. This may be an early indication of clothianidin resistance, but should be confirmed with further bioassays.

Insecticide selection pressure from agriculture is generally regarded as an important early driver of insecticide resistance in malaria vectors [30, 31]. According to the Insecticide Resistance Action Committee (IRAC), neonicotinoid resistance has been reported to at least 12 species of crop pest due to agricultural use in Asia, Europe and North America, with increased expression of various P450 genes implicated in several species [32–34]. Neonicotinoids are undoubtedly becoming more widely utilized for agricultural pest control in Africa. While usage statistics from sub-Saharan Africa are scanty, in 2018, there were a reported 45 neonicotinoid products registered in Cameroon (covering 4 types of neonicotinoid), 133 products registered in Tanzania (including 6 types), and > 70 products in Côte d’Ivoire for use against pests of cocoa, cotton, bananas, coffee and tomatoes [35–37]. Neonicotinoids are highly water soluble and can persist for months in aerobic soil, therefore making contamination of local water surfaces following agricultural use highly likely. Neonicotinoid residues were found to be a common occurrence in puddles located in maize fields with neonicotinoid seed treatment [38]. Such contamination of water sources may represent an important source of mosquito larval selection for neonicotinoid resistance in the future and should be monitored.

SumiShield™ 50WG received WHO PQ listing in 2017 and Fludora Fusion™ WP-SB in 2018. However, before these products can be used for IRS it is essential for baseline susceptibility testing to be conducted to ensure there is no existing resistance, either from agricultural use or cross-resistance through existing mechanisms. In a time where new insecticides for rotation strategies are desperately needed, it is vitally important for the WHO to implement timely multi-center studies to determine susceptibility protocols for new active ingredients so that guidance can be issued at the same time as a formulation receives PQ listing. A lack of susceptibility protocols for new active ingredients can result in delayed uptake due to NMCP (National Malaria Control Programme) or donor reluctance to utilize products where resistance monitoring can’t be effectively conducted.

## Conclusion

The protocol used in this study can serve as an interim diagnostic dose for determining susceptibility status, until the WHO completes multi-center studies to develop a suitable solvent system for bottle bioassays of neonicotinoids. However, the results obtained in our study show that more work is needed to determine a definitive diagnostic dose that can detect phenotypic resistance without resulting in overestimation of resistance. Results from this baseline study have shown no strong evidence for existing resistance to clothianidin in 16 countries in sub-Saharan Africa, including mosquitoes with multiple resistance mechanisms to pyrethroids, carbamates and organophosphates.

## Additional file

**Additional file 1.** Maximum and minimum temperature (°C) and relative humidity (%RH) during the 60 minutes testing and up to 7 day holding period & % Mortality of mosquitoes tested with the negative control (WHO papers treated with 2ml of distilled water) for up to 7 days after bioassay.

## Data Availability

The datasets used and/or analyzed during the current study are available from the corresponding author on reasonable request.

## Abbreviations

AChE: acetylcholinesterase
AIRS: Africa Indoor Residual Spraying Project
CS: capsule suspension
DDT: dichloro diphenyl trichloroethane
GPIRM: Global Plan for Insecticide Resistance Management
IRAC: Insecticide Resistance Action Committee
IRS: indoor residual spraying
ITN: insecticide-treated nets
LLIN: long-lasting insecticidal nets
LT: lethal time
LT_50_: lethal time predicted for 50% mortality to be reached
nAChR: nicotinic acetylcholine receptors
NMCP: National Malaria Control Programme
PMI: US President’s Malaria Initiative
PQ: prequalification listing
SCC: Sumitomo Chemical Company
WG: water dispersible granules
WHO: World Health Organization
WP: wettable powder
WP-SB: wettable powder in a water soluble bag
w/v: weight per volume
