# Supplementary material for: Susceptibility testing of Anopheles malaria vectors with the neonicotinoid insecticide clothianidin; results from 16 African countries, in preparation for indoor residual spraying with new insecticide formulations
Source: Malar J. 2019 Aug 1;18:264. doi: 10.1186/s12936-019-2888-6 (PMC6670198; doi:10.1186/s12936-019-2888-6)
Supplement: Supplementary file 1 — Additional file 1. Maximum and minimum temperature (°C) and relative humidity (%RH) during the 60 minutes testing and up to 7 day holding period & % Mortality of mosquitoes tested with the negative control (WHO papers treated with 2ml of distilled water) for up to 7 days after bioassay. [file 12936_2019_2888_MOESM1_ESM.docx]

**Additional Files**

**Table S1. Maximum and minimum temperature (°C) and relative humidity (%RH) during the 60 minutes testing and up to 7 day holding period.**

| **Country** | **Min-Max temperature °C** | **Min-Max humidity %RH** |
| --- | --- | --- |
| Tanzania | 21.0 - 27.1 | 47.9 - 93.0 |
| Madagascar | 23.9 - 28.5 | 73.0 - 83.0 |
| Nigeria | 25.9 - 28.9 | 80.7 - 90.7 |
| DR Congo | 24.0 - 25.0 | 75.0 - 82.0 |
| Uganda | 24.6 - 27.4 | 62.0 - 86.0 |
| Ethiopia | 25.0 - 28.0 | 56.0 - 92.0 |
| Liberia | 27.0 - 28.2 | 70.0 - 86.0 |
| Senegal | 27.0 - 28.4 | 71.0 - 96.0 |
| Zimbabwe | 19.0 - 26.0 | 54.0 - 68.0 |
| Burkina Faso | 25.7 - 27.0 | 54.0 - 74.0 |
| Ghana | 24.2 - 27.2 | 67.8 - 90.8 |
| Burundi | 23.0 - 28.0 | 66.5 - 83.8 |
| Mozambique | 22.0 - 25.9 | 69.0 - 88.0 |
| Kenya | 26.4 (mean) | 63.6 (mean) |
| Mali | 25.7-29.3 | 51.0-71.4 |
| Zambia | 27.8-30.2 | Not recorded |

**Table S2. % Mortality of mosquitoes tested with the negative control (WHO papers treated with 2ml of distilled water) for up to 7 days after bioassay.**

|  |  |  |  | **% Mortality** | | | | | | | |
| --- | --- | --- | --- | --- | --- | --- | --- | --- | --- | --- | --- |
| **Country** | **Site** | **Species** | **Tested** | **KD 60 mins** | **1 day** | **2 days** | **3 days** | **4 days** | **5 days** | **6 days** | **7 days** |
| Burkina Faso | Insectary | *An. gambiae s.s.* (Kisumu) | 25 | 0 | 0 | 0 | 0 | 0 | 0 | 0 | 0 |
| Burkina Faso | Insectary | *An. gambiae s.s.* (Kisumu) | 25 | 0 | 0 | 0 | 0 | 0 | 0 | 0 | 0 |
| Burkina Faso | Insectary | *An. gambiae s.s.* (Kisumu) | 25 | 0 | 0 | 0 | 4 | 4 | 4 | 4 | 4 |
| Burkina Faso | Insectary | *An. gambiae s.s.* (Kisumu) | 25 | 0 | 0 | 0 | 0 | 0 | 0 | 0 | 0 |
| Burkina Faso | Insectary | *An. gambiae s.s.* (Kisumu) | 25 | 0 | 0 | 0 | 0 | 0 | 0 | 0 | 0 |
| Burkina Faso | Insectary | *An. gambiae s.s.* (Kisumu) | 24 | 0 | 0 | 0 | 0 | 0 | 0 | 0 | 0 |
| Burkina Faso | Insectary | *An. gambiae s.s.* (Kisumu) | 25 | 0 | 0 | 0 | 0 | 0 | 0 | 0 | 0 |
| Burkina Faso | Insectary | *An. gambiae s.s.* (Kisumu) | 25 | 0 | 0 | 0 | 0 | 0 | 0 | 0 | 0 |
| Burkina Faso | Nouna | *An. gambiae s.l.* | 26 | 0 | 0 | 0 | 0 | 0 | 0 | 0 | 0 |
| Burkina Faso | Solenzo | *An. gambiae s.l.* | 24 | 0 | 0 | 0 | 0 | 0 | 0 | 0 | 0 |
| Burkina Faso | Kampti | *An. gambiae s.l.* | 25 | 0 | 0 | 0 | 0 | 0 | 0 | 0 | 0 |
| Burkina Faso | Gaoua | *An. gambiae s.l.* | 25 | 0 | 0 | 0 | 0 | 0 | 0 | 0 | 0 |
| Burkina Faso | Tiefora | *An. gambiae s.l.* | 25 | 0 | 0 | 0 | 0 | 0 | 0 | 0 | 0 |
| Burkina Faso | Mangodara | *An. gambiae s.l.* | 25 | 0 | 0 | 0 | 0 | 0 | 0 | 0 | 0 |
| Burkina Faso | Seguenega | *An. gambiae s.l.* | 24 | 0 | 0 | 0 | 0 | 0 | 0 | 0 | 0 |
| Burkina Faso | Kongoussi | *An. gambiae s.l.* | 24 | 0 | 0 | 0 | 0 | 0 | 0 | 0 | 0 |
| Burundi | Insectary | *An. gambiae s.s.* (Kisumu) | 50 | 0 | 0 | 0 | 0 | 0 | 0 | 0 | 0 |
| Burundi | Bubansa | *An. gambiae s.l.* | 50 | 0 | 2 | 6 | 6 | 6 | 6 | 6 | 6 |
| DR Congo | Insectary | *An. coluzzii* (Cameroon) | 25 | 0 | 0 | 0 | 0 | 0 | 0 | 0 | 0 |
| DR Congo | Kinshasa | *An. gambiae s.l.* | 25 | 4 | 4 | 4 | 4 | 4 | 4 | 4 | 4 |
| Ethiopia | Insectary | *An. arabiensis* (colony) | 49 | 0 | 4 | 4 | 6 | 10 | 10 | 12 | 16 |
| Ethiopia | Sodere | *An. gambiae s.l.* | 49 | 0 | 0 | 0 | 0 | 2 | 18 | 24* | 37* |
| Ghana | Insectary | *An. gambiae s.s.* (Kisumu) | 50 | 0 | 0 | 0 | 0 | 4 | 4 | 6 | 10 |
| Ghana | Insectary | *An. gambiae s.s.* (Kisumu) | 50 | 0 | 0 | 0 | 0 | 0 | 6 | 8 | 10 |
| Ghana | Insectary | *An. gambiae s.s.* (Kisumu) | 50 | 0 | 0 | 0 | 0 | 0 | 2 | 6 | 10 |
| Ghana | Kumbungu | *An. gambiae s.l.* | 100 | 1 | 1 | 2 | 6 | 7 | 8 | 11 | 12 |
| Ghana | Kumbungu | *An. gambiae s.l.* | 50 | 0 | 0 | 0 | 0 | 0 | 2 | 2 | 2 |
| Ghana | Gbullung | *An. gambiae s.l.* | 50 | 0 | 0 | 2 | 2 | 2 | 2 | 2 | 8 |
| Ghana | Gbullung | *An. gambiae s.l.* | 50 | 0 | 0 | 0 | 6 | 8 | 10 | 18 | 20 |
| Liberia | Frank Town | *An. gambiae s.l.* | 25 | 0 | 8 | 8 | 8 | 8 | 8 | 8 | 8 |
| Liberia | Frank Town | *An. gambiae s.l.* | 25 | 0 | 0 | 0 | 0 | 0 | 0 | 0 | 0 |
| Liberia | Frank Town | *An. gambiae s.l.* | 25 | 0 | 0 | 0 | 0 | 0 | 0 | 0 | 0 |
| Madagascar | Ambodifaho | *An. gambiae s.l.* | 50 | 0 | 0 | 0 | 0 | 0 | 0 | 0 | 0 |
| Madagascar | Vohitrambato | *An. gambiae s.l.* | 50 | 0 | 0 | 0 | 0 | 2 | 2 | 2 | 4 |
| Madagascar | Mahambo | *An. gambiae s.l.* | 50 | 0 | 2 | 2 | 2 | 4 | 4 | 4 | 4 |
| Madagascar | Vavatenina | *An. gambiae s.l.* | 50 | 0 | 0 | 0 | 0 | 0 | 2 | 4 | 4 |
| Madagascar | Lanivo | *An. gambiae s.l.* | 50 | 0 | 0 | 4 | 4 | 4 | 4 | 4 | 4 |
| Madagascar | Manambotra Sud | *An. gambiae s.l.* | 50 | 0 | 0 | 4 | 4 | 4 | 4 | 4 | 4 |
| Madagascar | Lopary | *An. gambiae s.l.* | 50 | 0 | 0 | 6 | 6 | 6 | 6 | 6 | 6 |
| Mozambique | Maganja da Costa | *An. funestus s.l.* | 50 | 0 | 6 | 6 | 8 | 10 | 18 | 22 | 22 |
| Mali | Insectary | *An. coluzzii* (Cameroon) | 50 | 0 | 0 | 0 | 0 | 0 | 0 | 0 | 0 |
| Mali | Insectary | *An. coluzzii* (Cameroon) | 50 | 0 | 0 | 0 | 0 | 0 | 0 | 0 | 0 |
| Mali | Insectary | *An. coluzzii* (Cameroon) | 50 | 0 | 0 | 0 | 0 | 0 | 10 | 10 | 10 |
| Mali | Insectary | *An. coluzzii* (Cameroon) | 50 | 0 | 0 | 0 | 0 | 0 | 0 | 0 | 0 |
| Mali | Djenne | *An. gambiae s.l.* | 48 | 0 | 0 | 0 | 0 | 0 | 0 | 0 | 0 |
| Mali | Mopti | *An. gambiae s.l.* | 50 | 0 | 0 | 0 | 4 | 6 | 6 | 6 | 6 |
| Mali | Bandiagara | *An. gambiae s.l.* | 50 | 0 | 0 | 0 | 0 | 0 | 0 | 0 | 0 |
| Mali | Bankass | *An. gambiae s.l.* | 50 | 0 | 0 | 0 | 0 | 0 | 0 | 0 | 0 |
| Nigeria | Insectary | *An. gambiae s.s.* (Kisumu) | 25 | 0 | 0 | 0 | 0 | 0 | 0 | 4 | 4 |
| Nigeria | Doma | *An. gambiae s.l.* | 25 | 0 | 0 | 0 | 0 | 0 | 4 | 4 | 8 |
| Nigeria | Keffi Nsuk | *An. gambiae s.l.* | 50 | 0 | 0 | 0 | 0 | 2 | 4 | 6 | 6 |
| Nigeria | Nasarawa Egon | *An. gambiae s.l.* | 50 | 0 | 0 | 0 | 2 | 2 | 2 | 6 | 10 |
| Nigeria | Nasarawa | *An. gambiae s.l.* | 50 | 0 | 0 | 4 | 6 | 8 | 8 | 8 | 8 |
| Nigeria | Panda Karu | *An. gambiae s.l.* | 50 | 0 | 0 | 2 | 6 | 8 | 8 | 8 | 8 |
| Senegal | Insectary | *An. coluzzii* (Cameroon) | 57 | 0 | 4 | 7 | 7 | 7 | 9 | 9 | 9 |
| Senegal | Dakar | *An. gambiae s.l.* | 55 | 0 | 0 | 4 | 4 | 5 | 5 | 7 | 7 |
| Tanzania | Insectary | *An. gambiae s.s.* (Kisumu) | 50 | 0 | 0 | 0 | 0 | 0 | 0 | 0 | 0 |
| Tanzania | Musoma Rural | *An. gambiae s.l.* | 50 | 0 | 0 | 0 | 0 | 0 | 0 | 0 | 0 |
| Tanzania | Musoma Rural | *An. gambiae s.l.* | 50 | 0 | 0 | 0 | 0 | 0 | 0 | 0 | 0 |
| Uganda | Insectary | *An. gambiae s.s.* (Kisumu) | 52 | 0 | 0 | 6 | 6 | 6 | 8 | 8 | 10 |
| Uganda | Insectary | *An. gambiae s.s.* (Kisumu) | 51 | 0 | 0 | 0 | 0 | 0 | 0 | 2 | 2 |
| Uganda | Totokidwe | *An. gambiae s.l.* | 50 | 0 | 0 | 2 | 2 | 2 | 2 | 2 | 6 |
| Uganda | Pokongo West | *An. gambiae s.l.* | 54 | 0 | 0 | 0 | 0 | 2 | 6 | 7 | 9 |
| Uganda | Tawojoko | *An. gambiae s.l.* | 27 | 0 | 7 | 11 | 11 | 11 | 11 | 11 | 11 |
| Uganda | Awanya | *An. gambiae s.l.* | 46 | 0 | 0 | 2 | 2 | 2 | 2 | 4 | 9 |
| Zambia | Insectary | *An. gambiae s.s.* (Kisumu) | 52 | 0 | 2 | 4 | 6 | 8 | 8 | 10 | 10 |
| Zambia | Insectary | *An. gambiae s.s.* (Kisumu) | 51 | 0 | 2 | 4 | 4 | 4 | 4 | 4 | 4 |
| Zambia | Miyambo | *An. funestus s.l.* | 53 | 0 | 4 | 8 | 8 | 8 | 13 | 13 | 15 |
| Zambia | Miyambo | *An. funestus s.l.* | 54 | 0 | 4 | 9 | 9 | 9 | 9 | 9 | 9 |
| Zambia | Shitambulli | *An. funestus s.l.* | 51 | 0 | 4 | 8 | 8 | 8 | 14 | 14 | 14 |
| Zambia | Shitambulli | *An. funestus s.l.* | 52 | 0 | 6 | 6 | 8 | 8 | 8 | 8 | 8 |
| Zimbabwe | Insectary | *An. arabiensis* (KGB) | 20 | 0 | 0 | 0 | 0 | 0 | 0 | 0 | 0 |
| Zimbabwe | Manjolo | *An. gambiae s.l.* | 20 | 0 | 0 | 0 | 0 | 0 | 0 | 0 | 5 |
| Zimbabwe | Chilonga | *An. gambiae s.l.* | 15 | 0 | 13 | 13 | 13 | 13 | 13 | 13 | 13 |

*Mortality was >20% in Sodere after 6 days, but mortality in wild *An. gambiae* s.l. did not increase after 5 days.
